# Therapeutic Strategy for Functional Metastatic Malignant Paraganglioma: A Case Report and Review of the Literature

**DOI:** 10.7759/cureus.60027

**Published:** 2024-05-10

**Authors:** Yousra Bennouna, Nadin Shawar Al Tamimi, Ganiou Adjade, Mohamed El Fadli, Ismail Essadi, Rhizlane Belbaraka

**Affiliations:** 1 Oncology, Centre Hospitalo-Universitaire (CHU) Mohammed VI, Marrakesh, MAR; 2 Medical Oncology, Centre Hospitalo-Universitaire (CHU) Mohammed VI, Marrakesh, MAR; 3 Medical Oncology, Ibn Sina Military Teaching Hospital, Marrakesh, MAR

**Keywords:** histology, prognosis, extra-adrenal paraganglioma, management, diagnosis

## Abstract

Paraganglioma is a rare neuroendocrine tumor that arises outside of the adrenal gland, typically originating from the chromaffin tissue of the sympathetic or parasympathetic ganglia. It can manifest at any age, with a peak incidence occurring between 40 and 50 years old. When the tumor secretes catecholamines, it is referred to as "functional." Currently, there is no standardized therapeutic approach. However, the management of metastatic forms is based on a systemic treatment with tri-chemotherapy.

Herein, we present the case of a young male patient with heavily metastatic functional malignant paraganglioma, which represents the first case managed in our department. After seven months of Somatuline treatment, our patient experienced disease progression. Subsequently, he received tri-chemotherapy comprising cyclophosphamide, vincristine, and dacarbazine, which proved to be suboptimal due to poor hematological tolerance and a progression-free survival of less than three months. In the third line of treatment, Sunitinib was administered, but the therapeutic response was poor, with clinical progression observed within two months, ultimately leading to the patient's demise at home. The overall survival was two years.

## Introduction

Pheochromocytomas (PHEOs) and paragangliomas (PGLs) are rare tumors of the autonomic nervous system and are generally benign [1]. Malignant forms are very rare, with their diagnosis based on the presence of loco-regional or distant metastases, occurring at an incidence that varies between 1/30,000 and 1/100,000 cases/inhabitants/year in the Caucasian population [2], with an average age at diagnosis of 40 years [1,3]. Diagnosis remains difficult.

There is no standardized therapeutic management. The most effective systemic treatment seems to be triple therapy with cyclophosphamide, vincristine, and dacarbazine (CVD).

We present, through a literature review, the first documented case of metastatic paraganglioma in our medical oncology department at the Mohammed VI University Hospital in Marrakesh.

## Case presentation

A 32-year-old male patient presented symptoms evolving over three years, consisting of persistent headaches, heart palpitations with a blood pressure peak at 220 mmHg for the systolic, and hyperhidrosis. The explorations revealed a type II diabetes of recent discovery. He was prescribed oral antidiabetic and antihypertensive medications for his severe hypertension.

**Figure 1:**
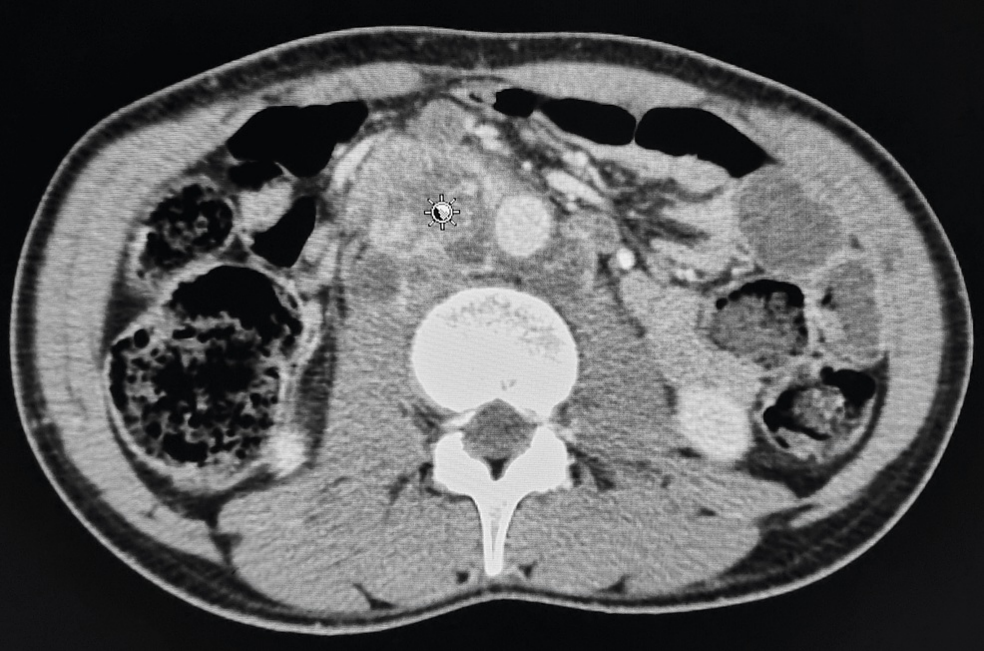
Multiple retroperitoneal masses, the largest measuring 45.2 x 42mm (pointer), envelope the large vessels, the right renal pedicle, and the left renal vein, compressing the inferior vena cava

The evolution was marked by the occurrence of persistent abdominal pain. A thoracic-abdominopelvic computed tomography (CT) scan, performed three months before the first consultation, showed a cluster of deep abdominal lymph nodes compressing the inferior vena cava (IVC), the largest of which measured 32x41mm, associated with bilateral right jugular-carotid and supra-clavicular lymphadenopathy (Figure 1).

**Figure 2:**
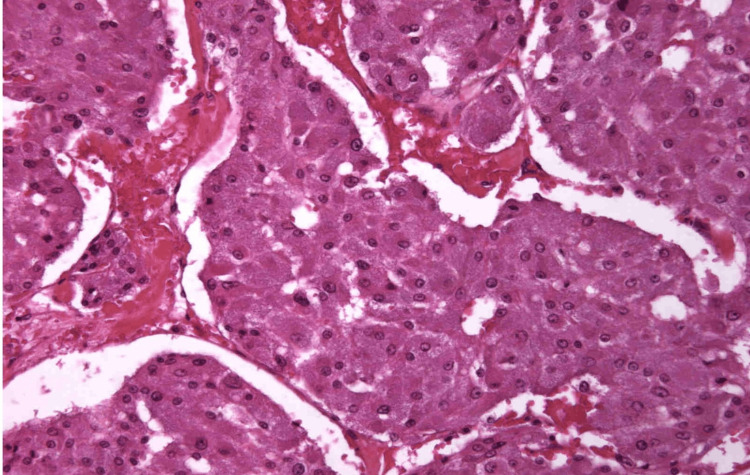
Epithelial cell population of endocrinoïd architecture

**Figure 3:**
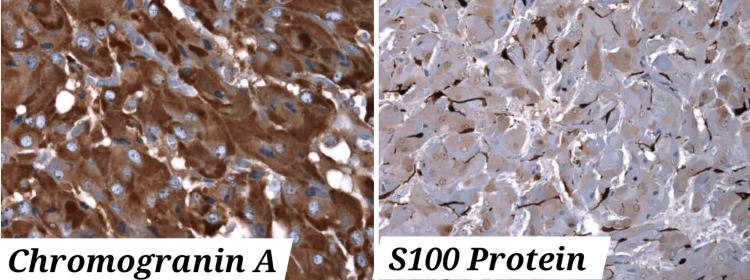
Chromogranin and S100 protein immunostain (with a Ki67 index upper to 20%)

A biopsy of the retroperitoneal adenopathy revealed, upon anatomopathological examination, a cell population with an endocrine architecture. Immunohistochemical analysis indicated compatibility with a paraganglioma, as evidenced by the expression of chromatogranin with a Ki67 index upper to 20% and anti-PS100 antibodies. It tested negative for anti-Pan cytokeratin and anti-CK7 antibodies (Figures 2, 3). 

A pretherapeutic baseline cervical-thoraco-abdominopelvic CT scan showed a tissue mass in the left cervicothoracic outlet, in contact with the posterior aspect of the carotid sheath, without signs of thrombosis. This was associated with secondary multifocal osteolytic lesions of the vertebral and costal bones, complicated by spinal cord compression at D6-D7. The patient underwent decompressive radiotherapy on vertebrae D3-D8.

On biological analysis, urinary methoxylated derivatives were four times the normal level, normetanephrines were 64 times the normal level, and metanephrines were negative. The patient received 13 injections of Lanreotide over seven months, with a clinical response during the first three months. However, by the seventh month, the evaluation revealed clinical progression of the disease, characterized by persistent vertebral pain, radiological progression with thrombosis of the IVC, and the appearance of two nodular lesions in the left gluteal region.

In a multidisciplinary tumor board, a second-line systemic treatment was indicated, consisting of a combination chemotherapy regimen of cyclophosphamide, vincristine, and dacarbazine. However, the patient received six nonoptimal cycles due to poor tolerance, experiencing hematological toxicity such as grade III anemia.

As a third-line treatment, he received SUTENT at the standard dose of 50mg daily in a 'four-week on-two week off.' After two months of treatment, the patient reported a deterioration of his general condition. He then died at home under unspecified circumstances, with an overall survival of two years.

## Discussion

Neuroendocrine neoplasms are rare and heterogeneous tumors of epithelial origin that arise from cells of the neuroendocrine system [4].

Pheochromocytomas (PHEOs) and paragangliomas (PGLs) are rare neuroendocrine tumors that arise from chromaffin cells. PHEOs originate from the adrenal medulla, while PGLs originate from the extra-adrenal sympathetic or parasympathetic nervous system [1]. Extra-adrenal sympathetic PGLs represent only 10% of all paragangliomas [3].

The functional paraganglioma secretes excess catecholamines, which manifest clinically as paroxysmal hypertension, headaches, sweating, and palpitations (Menard's triad), and this applies to our patients.

The diagnosis is based on histological analysis. Macroscopically, it appears as a huge, rounded tumor, encapsulated, highly vascularized, and with a firm and elastic consistency [5]. Immunohistochemistry is essential for confirmation due to the challenging nature of the diagnosis. There are no histologic criteria to distinguish benign from malignant tumors. Malignancy is defined by the presence of metastases in sites where paraganglionic tissue is normally absent. The most frequently metastatic sites are lymph nodes (70%), skeletons (68%), livers (46%), and lungs (39%) [6].

Biological markers such as 24-hour urine fractionated (nor-)metanephrines, plasma-free (nor-)metanephrines, methoxytyramine, and plasma or urine chromogranin are the most sensitive, and they are often elevated to values that correlate with tumor mass [7].

Computed tomography scans and magnetic resonance imaging (MRI) can localize and stage the disease but with low specificity. Metaiodobenzylguanidine (MIBG) scintigraphy labeled with iodine 123 or 131 has high sensitivity and specificity. It is useful for detecting metastases when radiometabolic treatment with 131 iodine-MIBG is considered.

The identification of a constitutional mutation in the succinate dehydrogenase B subunit (SDHB) gene is considered a risk factor for malignancy [8] and is associated with a poor prognosis. In the metastatic setting, the five-year overall survival was 36% for patients with an SDHB mutation and 67% in the absence of this mutation [6]. 18F-fluorodeoxyglucose positron emission tomography (FDG-PET) is recommended in metastatic PGL with a succinate dehydrogenase mutation.

Due to the rarity of this group of tumors, there is no standardized therapeutic strategy, necessitating a multidisciplinary approach to treatment. Systemic therapy is typically recommended for metastatic and/or functional tumors. In the population with functional tumors, somatostatin analogs are commonly employed as first-line therapy.

Somatostatin is a hormone secreted by the hypothalamus, pancreatic, and intestinal D-cells. This hormone exerts a wide variety of effects as it acts on diverse populations of target cells. It operates with a dual action, both central and peripheral. At the hypothalamic-pituitary level, somatostatin regulates the secretion of growth hormone (GH) by inhibiting it. At the intestinal level, somatostatin, whether endogenously produced or administered exogenously, primarily exerts an inhibitory effect on the secretion of intestinal peptide hormones, such as vasoactive intestinal peptide (VIP), gastrin, cholecystokinin, gastric inhibitory polypeptide (GIP), and secretin, mainly through local paracrine mechanisms. There are currently five types of somatostatin receptors (SSTR), denoted as SSTRs one through five, present on the surface of cells throughout the body, including chromaffin cells. Its anti-proliferative action is mediated through the SSTR2 receptor, which is activated by somatostatin analogs such as lanreotide or octreotide. Somatuline is the extended-release formulation of lanreotide. It functions by inducing cell cycle arrest, or apoptosis, through its inhibitory action on the secretion of growth factors [9].

Iodine-131 metaiodobenzylguanidine (131I-MIBG), a radiopharmaceutical agent utilized for scintigraphic localization of PHEO/PGL, has been employed for treating metastatic PHEO/PGL expressing the norepinephrine transporter (NET) in tumor cell membranes. In a meta-analysis by van Hulsteijn et al., 17 studies comprising 243 patients with malignant PHEO/PGL treated with 131I-MIBG therapy were reviewed. The analysis revealed stable disease in 52% of patients and a partial hormonal response in 40%. Reported five-year survival rates ranged from 45 to 64%. The mean progression-free survival, based on two studies, was 23 and 28 months, respectively. 131I-MIBG may offer clinical benefits in patients with locally advanced or metastatic PHEO/PGL [10].

Triple therapy with cyclophosphamide, vincristine, and dacarbazine appears to be the most effective [11], with a complete response rate of 4%, a partial response rate of 37%, and disease stability of 14%, according to a meta-analysis [12]. Temozolomide is an effective antitumor agent in patients with metastatic PGLs, especially in cases of SDHB mutations [13].

Anti-angiogenic therapies may be considered as treatment options since PGLs are highly vascularized tumors [14,15]. Sunitinib has also been evaluated in a randomized phase II trial involving 25 patients, with 13% of patients achieving a partial response and 70% experiencing stable disease [16].

Patients with advanced, rare cancers often face a poor prognosis and limited treatment options. Immunotherapy has demonstrated effectiveness in various cancer types, particularly in rare tumors, owing to its capability to target the immune system rather than the tumor itself. Pembrolizumab, based on its rate of durable objective responses and favorable safety profile, underwent evaluation in this indication through a phase II trial, leading to its approval by the Food and Drug Administration (FDA) in 2018 [17]. In the trial, nine patients with PGL were included, and after 27 weeks of follow-up, seven patients were evaluable (78%), with three patients showing no progression. Regarding the evaluation of objective response and clinical benefit, six patients (75%) were found to have stable lesions, while two patients progressed. There were no complete or partial responses, but six patients experienced clinical benefit.

Everolimus treatment has been assessed in patients with metastatic PHEO/PGLs [18] in a phase II study involving seven patients, five of whom exhibited stable disease. The median progression-free survival and duration of treatment for these patients were almost four months [19].

Our patient was followed for functional multi-metastatic PGL, presenting with concurrent diabetes and hypertension. This case represents the first instance treated at our center. The patient experienced initial tumor progression after seven months of Lanreotide. As a second-line option, standard chemotherapy was administered, consisting of the combination of cyclophosphamide, vincristine, and dacarbazine (CVD), but the progression-free survival did not exceed three months, and the hematological tolerance was very poor. Therefore, the treatment was suboptimal, which may account for this rapid progression. Subsequently, in the third line of treatment, the patient received targeted therapy with Sunitinib. However, the therapeutic response after two months of treatment was similarly poor, with clinical progression ultimately leading to the patient's demise.

## Conclusions

Paragangliomas are rare and aggressive neuroendocrine tumors that develop in the parasympathetic nervous system. Their diagnosis is challenging, and their management requires a multidisciplinary approach. In metastatic forms, systemic treatment with tri-chemotherapy remains the standard approach. The therapeutic response to chemotherapy and sunitinib initiated in our patient was poor, with progression-free survival (PFS) and overall survival (OS) not exceeding three months and two years, respectively. 
